# Insight into Enhanced Microwave Heating for Ammonia Synthesis: Effects of CNT on the Cs–Ru/CeO_2_ Catalyst

**DOI:** 10.1021/acsami.3c00132

**Published:** 2023-05-11

**Authors:** Alazar Araia, Yuxin Wang, Changle Jiang, Sean Brown, Ashley Caiola, Brandon Robinson, Wenyuan Li, Jianli Hu

**Affiliations:** Department of Chemical and Biomedical Engineering, West Virginia University, Morgantown, West Virginia 26506-6201, United States

**Keywords:** ammonia synthesis, microwave heating, Cs−Ru/CeO_2_ catalyst, carbon nanotubes, mechanically mixed catalyst, electrical conductivity, synergistic effect

## Abstract

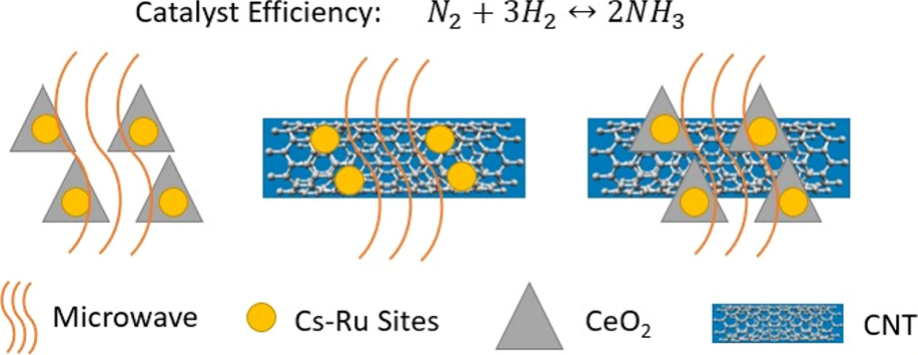

Ammonia is emerging as a potential decarbonized H_2_ energy carrier when produced from renewable energy. The on-site production of liquid ammonia from stranded renewable energy can solve the current energy transportation challenges. The employment of microwave technology can produce the desired ammonia product at milder conditions with the supply of intermittent renewable energy sources. Our previous studies have indicated that the Cs–Ru/CeO_2_ catalyst is a promising catalyst for microwave-driven ammonia synthesis. In this study, the Cs–Ru/CeO_2_ catalyst mechanically mixed with carbon nanotubes (CNT) and chemically synthesized using coprecipitation and a hydrothermal method is investigated systematically at low temperatures and atmospheric pressure for microwave-assisted ammonia synthesis. Additionally, the combination of two Ru-based catalysts (Cs–Ru/CeO_2_ and Cs–Ru/CNT) is studied as well. Mechanical mixing of Cs–Ru/CeO_2_ with CNT exhibited superior activity as compared to the chemically synthesized Cs–Ru/CeO_2_-CNT catalyst. Besides the enhancement in dielectric property, the probable synergistic effect leads to increased interfacial polarization at the interface of the mechanically mixed catalyst, improving the overall heating and ammonia production rate. Moreover, the combined Ru-based catalyst also exhibited higher activity as compared to their individual activity toward ammonia synthesis. Numerous characterization techniques were performed, including thermal imaging camera and dielectric measurements, to better understand microwave interaction with the composite catalysts.

## 1 Introduction

Ammonia is one of the greatest innovations of the 20th century, with extensive applications from fertilizers to intermediates for nitrogen-containing chemicals and pharmaceuticals.^1,2^ Annually, more than 242 million tons of ammonia is produced globally and support approximately 27% of the world’s population.^1,3,4^ Additionally, in recent years, ammonia is evolving as a major decarbonized H_2_ energy courier due to its high energy density.^4−6^ According to the U.S. Department of Energy, ammonia is considered a carbon-neutral energy source when produced from renewable energy.^7,8^ Industrial large-scale ammonia production is through the Haber–Bosch process operating at high pressures (150–300 bar) and high temperatures (400–500 °C) over iron-based catalysts. This industrial process is capital and energy intensive, and with the rapid increase in demand, there is an urgency to produce ammonia under milder conditions. Numerous alternative approaches have been proposed to the current industrial ammonia synthesis process to mitigate the cost intensive production.^9,10^ Microwave technology and microwave susceptor catalysts can yield the desired ammonia product under milder conditions.^5,11^ Besides mild reaction conditions, microwave technology can operate on intermittent renewable energy sources and is capable of accommodating small-medium scale plants. The unique advantage of microwaves relies on their swift response time with rapid heating and cooling in addition to quick start-up/shutdown, making microwaves a potential candidate for renewable ammonia and H_2_ production.^5,10,11^

Microwave irradiation is waves of energy converted to heat energy depending on the type of interaction with the associated material.^5^ Microwave heating has several advantages over conventional thermal heating, including selective and rapid heating. Microwaves selectively heat active sites on the catalyst surface as compared to conventional thermal heating of the bulk catalyst through conduction or convection heating.^9,12^ This microwave-selective heating in heterogeneous catalysis significantly reduces various reaction conditions like temperature, pressure, time, and activation energy.^5,13^ However, selective and volumetric heating also lead to non-uniform heating that may reduce the catalyst utilization. The selection of dielectric material is highly important, as microwave-selective heating is most proficiently driven due to the direct absorption and transfer of microwave energy within the catalyst system.^14,15^ Carbon materials are best suited for microwave heating attributed to the delocalized π electrons from sp^2^-hybridized carbon networks. The presence of these free electrons permits carbon nanotubes (CNT) to absorb microwave energy significantly and to be heated efficiently by incoming microwaves.^12^ In microwave-assisted ammonia synthesis, electromagnetic irradiation selectively heats the catalyst through dielectric loss or Debye-type loss heating, activating N_2_ and H_2_ molecules over the catalyst surface.^9,10^ This activated N_2_ and H_2_ species combine to synthesize the anticipated ammonia product. As the reaction is carried out on the catalyst surface, the development of microwave-sensitive catalysts is extremely vital for improved catalytic activity under microwave irradiation.

Catalyst development is a continuous process to further optimize the catalytic performance while increasing production. Therefore, it is essential to develop a microwave-susceptible catalyst with high productivity and stability toward ammonia synthesis. Wang et al. reported that Cs–Ru/CeO_2_ is a promising catalyst for microwave-assisted ammonia synthesis.^10^ According to his study, the Cs–Ru/CeO_2_ catalyst exhibited higher activity as compared to the Cs–Ru/MgO catalyst at the same reaction conditions toward ammonia synthesis under microwave irradiation. He designated to the small size of Ru species and the reversible oxidation state of ceria, making it a favorable support for ammonia synthesis.^10^ Moreover, our previous studies also reported on the durability of the Cs–Ru/CeO_2_ catalyst for microwave-assisted ammonia synthesis. Based on our findings, we can say that the Cs–Ru/CeO_2_ catalyst is stable and exhibited no loss in activity nor deactivation under microwave irradiation.^16,17^

To further improve the catalytic activity under microwave irradiation, we need to increase the susceptivity of the catalyst. A dielectric catalyst is highly important to convert electromagnetic radiation to heat, allowing the catalyst to initiate reaction under microwave irradiation.^18^ Carbon materials, especially CNT, are a very good microwave absorbent and can convert electromagnetic irradiation to heat easily.^12,19^ Carbon materials in nano- and microstructures have a promising application in catalysis.^14^ In the 1990s, KBR (Kellogg Advanced Ammonia Process) improved ammonia production through the development of a Ru catalyst supported on graphite carbon.^20,21^ Since then, much of the research attention was focused on Ru, especially for ammonia synthesis. The utilization of CNT and other carbon materials can improve the dielectric loss factor, facilitating heating rates and other mechanical properties.^14,22^ They can also enhance microwave heating and electrical conductivity, improving the overall catalytic activity.^12,22^ Due to this unique characteristic of carbon materials, especially CNT, it has gained enormous interest, particularly in microwave-assisted heterogeneous catalysis.^14^

In this paper, the effects of CNT on ammonia synthesis under microwave condition for a Cs–Ru/CeO_2_ catalyst are comprehensively studied. The Cs–Ru/CeO_2_ catalyst mechanically mixed with CNT and chemically synthesized using the coprecipitation method and the hydrothermal method is investigated systematically. In addition, the combination of two Ru-based catalysts (i.e., Cs–Ru/CeO_2_ and Cs–Ru/CNT) is also studied as well. Different characterization techniques, including a thermal imaging camera, were utilized to visualize heat distribution under microwave irradiation. The main novelty or innovation of this work is in optimizing the microwave sensitivity of the Cs–Ru/CeO_2_ catalyst to further enhance the ammonia production.

## 2 Experimental Section

### 2.1 Catalyst Preparation

Cerium (III) nitrate hexahydrate [Ce (NO_3_)_3_·6H_2_O, 99% trace metal basis], cerium oxide (CeO_2_ 50 nm nanopowder, >99.95% purity), ruthenium(III) nitrosyl nitrate (Ru [NO][NO_3_]_3_, Ru 31.3% min), and cesium nitrate (CsNO_3_, 99.8% metal basis) were purchased from Sigma-Aldrich. Multiwalled carbon nanotubes [MWCNT, 20–30 nm outer diameter (OD) with 10–30 μm length, surface area > 110 m^2^/g] were supplied from cheap tubes.

The Cs–Ru/CeO_2_ catalyst is prepared using incipient wetness impregnation. CeO_2_ (50 nm) support was impregnated with 2 wt % CsNO_3_ and 4 wt % Ru [NO][NO_3_]_3_, stirred for 6 h, dried in a drying oven for 12 h, and finally calcinated in air at 550 °C for 6 h. The Cs–Ru/CNT catalyst is prepared with the same impregnation method, while CNT support was adopted instead of CeO_2_.

For the mechanically mixed catalyst, a given mass of Cs–Ru/CeO_2_ catalyst was mixed mechanically with CNT at a 3:1 weight ratio using a mortar and pestle. 0.6 g of Cs–Ru/CeO_2_ catalyst is mixed with 0.2 g CNT mechanically and is referred as Cs–Ru/CeO_2_ + CNT_MM_.

The Cs–Ru/CeO_2_-CNT catalyst was synthesized using the coprecipitation method. 4 wt % Ru [NO][NO_3_]_3_ and 2 wt % CsNO_3_ were dissolved in ethanol. A 3:1 ratio of CeO_2_ to CNT is added simultaneously under continuous stirring. The mixture is then dried in a drying oven at 80 °C for 12 h. Lastly, it was calcined under the flow of nitrogen at 550 °C for 6 h. The catalyst prepared using the coprecipitation method is referred as Cs–Ru/CeO_2_-CNT_Cp_.

A hydrothermal process is also applied to synthesize CeO_2_-CNT catalyst support having a 3:1 ratio, respectively. 0.04 g NaOH and 4.34 g Ce (NO_3_)_3_·6H_2_O were dissolved separately in 10 mL of distilled water. The NaOH solution was then added to the cerium precursor under continuous stirring. CNT was dissolved in 10 mL of ethanol separately. The CNT solution was then added to the mixed cerium salt solution, and the mixture was stirred for 1 h. Afterward, the solution was transferred into an autoclave and kept at 180 °C for 3 h. After the treatment, the precipitate was washed with deionized water and ethanol until pH = 7, then dried in an oven overnight at 80 °C. Finally, the dried solids were calcined under the flow of nitrogen at 550 °C for 6 h. The synthesized CeO_2_-CNT support is then impregnated with 2 wt % CsNO_3_ and 4 wt % Ru [NO][NO_3_]_3_, stirred for 6 h, dried in an oven for 12 h, and finally calcinated at 550 °C under the flow of nitrogen for 6 h. The catalyst prepared using the hydrothermal method is referred to as Cs–Ru/CeO_2_-CNT_Hy_.

### 2.2 Catalyst Testing

The catalyst’s performance was tested in a fixed-bed reactor made of 8 mm inner diameter (ID) and 12 mm OD quartz tubes for ammonia synthesis. A given mass of catalyst 60–100 mesh was loaded into a quartz tube. The reactor tube was positioned in a variable frequency microwave reactor system (Lambda Technology, MC1330-200). The microwave reactor consists of two IR sensors. One is used to measure reactor tube temperature, and the second sensor is used to measure catalyst surface temperature. 75 vol. % H_2_ and 25% vol. N_2_ under 6000 mL/g_cat_ h gas hourly space velocity (GHSV) were flown to investigate the catalytic activity toward ammonia synthesis. N_2_ and H_2_ inlet gases were ultra-high purity grade (UHP, 99.999%), purchased from Airgas, Inc.

The catalyst bed was initially ramped to the reaction temperature of 260 °C and held for 120 min. Subsequently, the temperature was raised to 300, 340, and 360 °C at 30 min intervals. The final ammonia product was analyzed using a four-channel Micro-GC (Inficon 3000). All the catalyst activity tests were conducted under a microwave frequency of 5.850 GHz.

### 2.3 Characterization

X-ray diffraction (XRD) characterization of the powder samples was performed using PANalytical X’Pert Pro (PW3040) with Cu Kα radiation set to 45 kV and 40 mA. The scans were taken from 10 to 100° at a scan rate of 5 °/min.

Chemisorption with carbon monoxide (CO) as the adsorbate was performed to determine Ru particle size and metal dispersion. Autochem HP 2950 was utilized to carry out the measurements at 35 °C. The catalyst powder samples were reduced under the flow of H_2_ at 400 °C for 30 min prior to analysis. Finally, chemisorption data were used to calculate Ru particle size and dispersion.

Hydrogen temperature-programed reduction (H_2_-TPR) was performed in a Micromeritics Autochem HP 2950 instrument. Prior to measurement, the catalyst powder sample was pretreated at 150 °C for 60 min under the flow of N_2_ (30 mL/min). After the sample was cooled down to 100 °C, the gas flow was switched to 10% H_2_ in argon (30 mL/min). Finally, the powder sample was heated to 900 °C at a temperature ramp of 10 °C/min. The signal attained from the TCD was used to determine H_2_ consumption.

A transmission electron microscope (JEOL JEM-2100 LaB6) was utilized to image the powder catalyst. The Gatan ES500W camera was used with magnification ranging from 200 K (100 nm) to 600 K (20 nm).

An infrared thermal imaging camera (FLIR model number A6261) was utilized to visualize the microwave catalyst bed. The thermal imaging camera was situated 0.2 m from the quartz waveguide port.

Electrical conductivity measurement was studied comprehensively. The dry-pressed catalyst samples in the form of pellets were coated with gold paste (Nexceris Inc.) on both sides. The gold wire was used as the lead. The paste was dried in an oven at 120 °C overnight in the air. A standard four-probe DC configuration was used to measure the electrical conductivity of these samples. During measurement, a 0.1 mA current was supplied by the Keithley Sourcemeter 2400, and the voltage was measured by the Keithley Nanovoltmeter 2182A. The samples were measured in a temperature range of 40–300 °C in a tube furnace with a 7 °C/min ramping rate. The gas composition was controlled by mass flow controllers (Alicat Scientific).

Dielectric measurement was conducted using a Keysight P5002A vector network analyzer having a 7 mm by 3.12 cm air-line (Maury Microwave model number 2653S3.12) between 100 MHz and 9 GHz. The Keysight 85091C electronic calibration model was utilized for calibration on the autocalibration setting. The catalyst powder samples were included in a paraffin wax (Sigma-Aldrich, mp 53–58 °C) matrix at 10% volume loading, homogenized, and cast into a plug adhering to the process in Tempke et al.^23^ To separate the dielectric properties of the catalyst powder sample and matrix, Landau–Lifshits–Loonyenga (eq 1) was used.
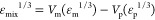
1where ε_mix_ is the measured property; *V*_m_ is the volume of the matrix; ε_m_ is the dielectric property of the matrix; *V*_p_ is the volume of the particle; ε_p_ is the dielectric property of the particle.

## 3 Results and Discussion

### 3.1 Catalytic Performance Comparison

**Figure 1:**
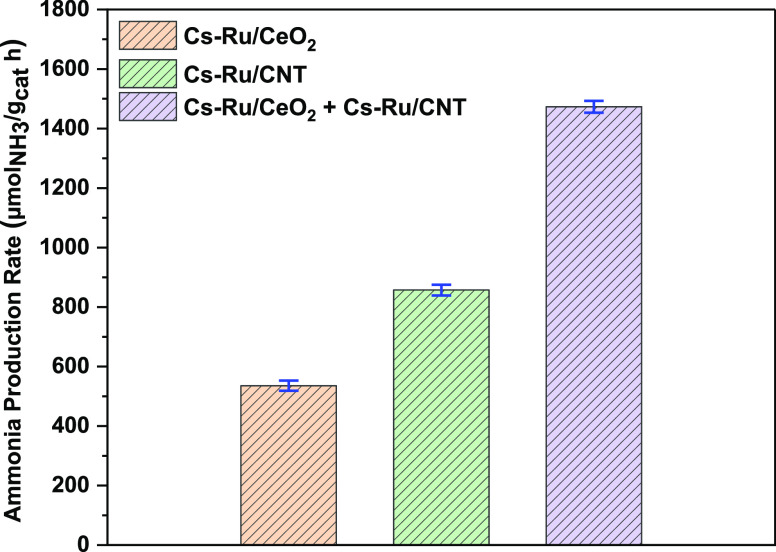
Catalytic activity for Cs–Ru/CeO_2_, Cs–Ru/CNT, and combined Ru-based catalysts at 260 °C toward microwave-assisted ammonia synthesis.

The catalytic activities of Cs–Ru/CeO_2_, Cs–Ru/CNT catalysts, and the combination of both ruthenium-based catalysts are studied in microwave-assisted ammonia synthesis. All the catalysts were total loaded with 2 wt % Cs and 4 wt % Ru. The catalytic activity of the Cs–Ru/CeO_2_ catalyst showed the highest conversion of 535 μmol NH_3_/g_cat_ h ammonia production rate at 260 °C. In contrast, Cs–Ru/CNT exhibited the highest conversion of 857 μmol NH_3_/g_cat_ h ammonia production rate at 260 °C, as shown in Figure 1. Herein, the ammonia synthesis activity of Cs–Ru/CeO_2_ is considerably lower than that of the Cs–Ru/CNT catalyst. However, when both catalysts, Cs–Ru/CeO_2_ and Cs–Ru/CNT (1:1 weight ratio), are combined mechanically using a mortar and pestle, the catalytic activity increased, reaching 1474 μmol NH_3_/g_cat_ h ammonia production rate. The combination of both Ru-based catalysts is much higher than that of the individual Cs–Ru/CNT and Cs–Ru/CeO_2_ catalysts. This suggests the individual synergistic effect of the two catalysts is higher when combined as compared to their individual catalytic activity, resulting in higher ammonia production.^24−26^ This synergy is ascribed to improved microwave heating and electrical conductivity when combined, facilitating the transfer of electrons to the Ru surface, expediting N_2_ dissociation, and resulting in higher ammonia production.^10,24,26^

**Figure 2:**
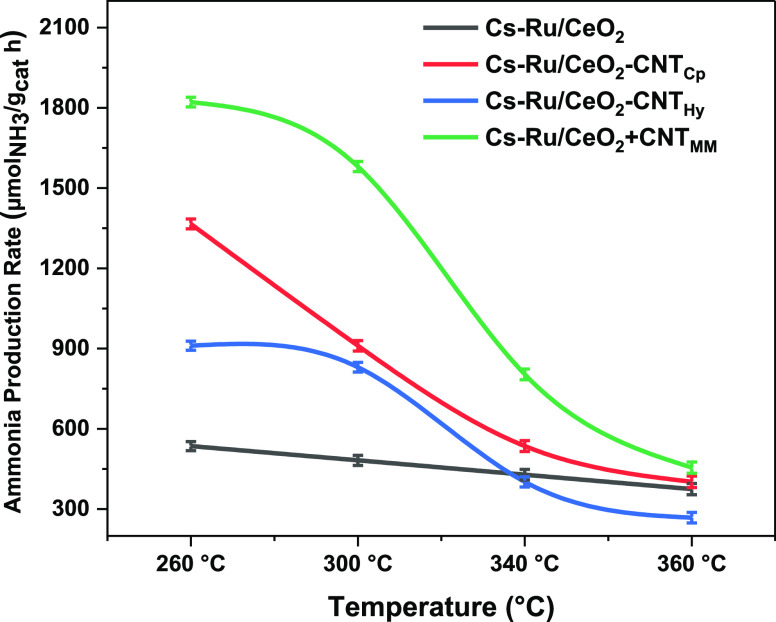
Ammonia production for Cs–Ru/CeO_2_, Cs–Ru/CeO_2_ + CNT_MM_, Cs–Ru/CeO_2_-CNT_Cp_, and Cs–Ru/CeO_2_-CNT_Hy_ catalysts toward ammonia synthesis.

The Cs–Ru/CeO_2_ catalyst mechanically mixed with CNT and the Cs–Ru/CeO_2_ catalyst chemically synthesized with CNT using coprecipitation and hydrothermal methods are studied for microwave-assisted ammonia synthesis, as shown in Figure 2. The Cs–Ru/CeO_2_ catalyst performance test is added for comparison purposes. When CNT was mechanically mixed with Cs–Ru/CeO_2_ catalyst (Cs–Ru/CeO_2_ + CNT_MM_), the catalytic activity increased dramatically, reaching 1822 μmol NH_3_/g_cat_ h ammonia production rate at 260 °C. To understand the increase in ammonia production rate when CNT was mixed mechanically with the Cs–Ru/CeO_2_ catalyst, the Cs–Ru/CeO_2_ catalyst with CNT was synthesized using the coprecipitation method and the hydrothermal method. The highest activity obtained for the Cs–Ru/CeO_2_-CNT_cp_ catalyst synthesized using the coprecipitation method was 1367 μmol NH_3_/g_cat_ h ammonia production rate and 910 μmol/NH_3_/g_cat_ h for the hydrothermally synthesized Cs–Ru/CeO_2_-CNT_Hy_ catalyst at 260 °C. Moreover, in all the catalysts investigated, mechanically mixed catalyst (Cs–Ru/CeO_2_ + CNT_MM_) exhibited superior activity as compared to Cs–Ru/CeO_2_-CNT_Hy_ and Cs–Ru/CeO_2_-CNT_Cp_ catalysts. The highest activity obtained for the mechanically mixed (Cs–Ru/CeO_2_ + CNT_MM_) catalyst under microwave irradiation is ascribed to an increase in the dielectric property.^19,27,28^ Besides the enhancement in dielectric property, the probable synergistic effect of the two catalysts leads to an increase in interfacial polarization at the interface of the mechanically mixed catalyst, improving the overall heating and ammonia production rates.^18,24^ Additionally, owing to CNT’s excellent microwave susceptibility and conductivity, it enhanced the electrical conductivity of the Cs–Ru/CeO_2_ catalyst, easing the transfer of electrons from support and promoter to the Ru surface, increasing the ammonia production.^12,22,29,30^ In all the catalysts investigated, the activity decreased as temperature increased. The decrease in ammonia production as temperature increases is mostly related to the exothermic nature of the ammonia synthesis reaction, which is not favorable at high temperatures.^31^

### 3.2 Catalyst Characterization

#### 3.2.1 XRD Measurement

**Figure 3:**
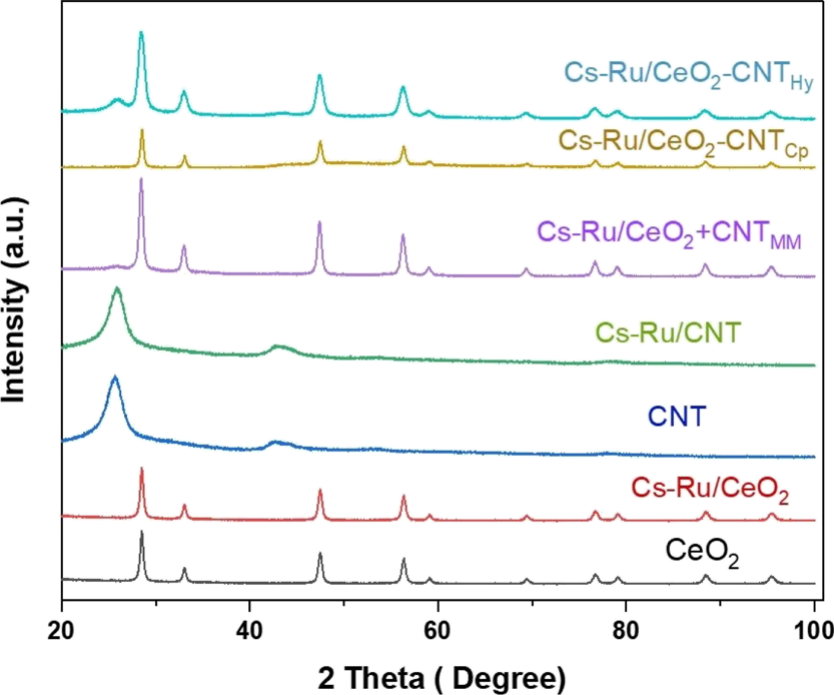
XRD diffraction peaks for Cs–Ru/CeO_2_, Cs–Ru/CNT, Cs–Ru/CeO_2_ + CNT_MM_, Cs–Ru/CeO_2_-CNT_Cp_, and Cs–Ru/CeO_2_-CNT_Hy_ catalysts.

An XRD study was performed to analyze the crystalline phase and structural property for all Ru-based catalysts. The diffraction peaks for blank CeO_2_ and CNT are added for reference, as shown in Figure 3. The CNT diffraction peak is detected for both the Cs–Ru/CNT catalyst and the hydrothermally synthesized Cs–Ru/CeO_2_-CNT_Hy_ catalyst, while a strong cerium oxide peak is observed for all the ceria-based catalysts. However, it was very hard to detect a CNT peak for the coprecipitation (Cs–Ru/CeO_2_-CNT_Cp_) and mechanically mixed (Cs–Ru/CeO_2_ + CNT_MM_) catalysts associated with high dispersion. Moreover, in all the catalysts studied, the Cs and Ru diffraction peaks were not detected. This indicates Cs and Ru are highly dispersed, having small particle sizes beyond the detection limit of the XRD.

#### 3.2.2 CO Chemisorption Measurement

CO chemisorption was conducted to study Ru particle size and dispersion on both CeO_2_ and CNT support and on the composite of both supports, either mechanically mixed or chemically synthesized, as shown in Table 1. The Cs–Ru/CeO_2_ catalyst was added for reference. When CNT was mechanically mixed with the Cs–Ru/CeO_2_ catalyst (Cs–Ru/CeO_2_ + CNT_MM_), no measurable difference in Ru particle size and dispersion was observed compared to its precursor Cs–Ru/CeO_2_ catalyst. However, when CNT were applied as a support (Cs–Ru/CNT), the highly dispersed and smaller Ru particle size was analyzed as compared to the metal-oxide support catalyst (Cs–Ru/CeO_2_), suggesting that the CNT support contributed to the formation of small-sized Ru species with high dispersion. Additionally, in the chemically synthesized catalyst using the coprecipitation method (Cs–Ru/CeO_2_-CNT_Cp_), Ru particle size was slightly bigger with lower dispersion as compared to the Cs–Ru/CNT catalyst, while the hydrothermally synthesized Cs–Ru/CeO_2_-CNT_Hy_ catalyst exhibited the smallest Ru particle size with high dispersion as compared to all the catalysts studied. The result obtained from CO chemisorption suggests CNT support contributed to improved dispersion and the formation of small Ru particle size. Yin et al. suggested that the nano-sized and high surface area of the CNT limits the growth of Ru over CNT while increasing dispersion.^32^

**Table 1:** CO Chemisorption for Cs–Ru/CeO_2_, Cs–Ru/CNT, Cs–Ru/CeO_2_ + CNT_MM_, Cs–Ru/CeO_2_-CNT_Cp_, and Cs–Ru/CeO_2_-CNT_Hy_ Catalysts for Ammonia Synthesis

catalyst	Ru particle size (nm)	Ru dispersion (%)	NH_3_ production rate at 260°C (μmol NH_3_/g_cat_ h)
Cs–Ru/CeO_2_	8.2	16.2	535
Cs–Ru/CNT	4.9	26.5	857
Cs–Ru/CeO_2_ + CNT_MM_	8.2	16.6	1822
Cs–Ru/CeO_2_-CNT_Cp_	5.3	24.8	1367
Cs–Ru/CeO_2_-CNT_Hy_	4.2	31.8	910

#### 3.2.3 H_2_-TPR Measurement

**Figure 4:**
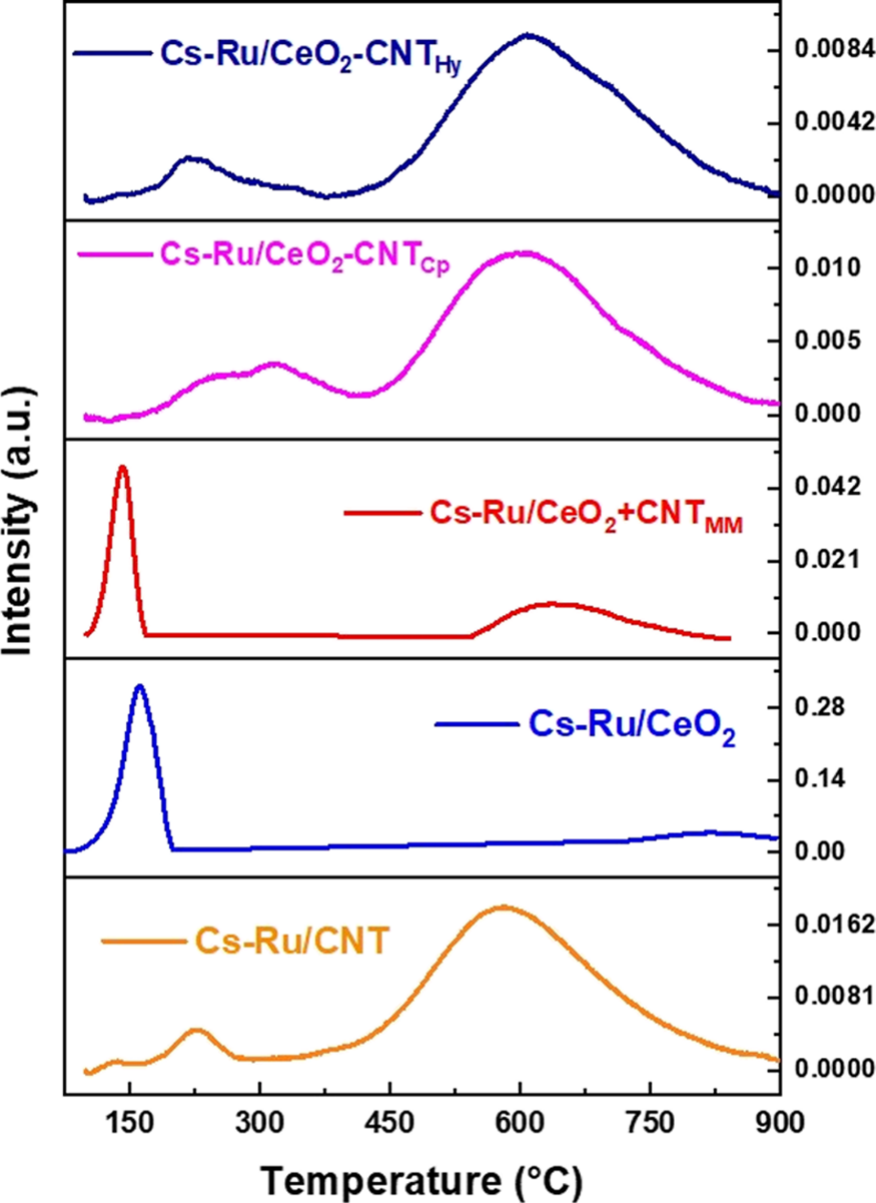
H_2_-TPR for Cs–Ru/CeO_2_, Cs–Ru/CNT, Cs–Ru/CeO_2_ + CNT_MM_, Cs–Ru/CeO_2_-CNT_Cp_, and Cs–Ru/CeO_2_-CNT_Hy_ catalysts.

Temperature-programed reduction (H_2_-TPR) was conducted to study the effect of CNT on the reducibility of Ru particles. Ru reduction temperature on CNT and cerium oxide support is investigated as well for a better understanding of the composite catalysts. As shown in Figure 4, two peaks are observed; the low-temperature peak is attributed to the reduction of the Ru species, while the second peak is attributed to the reduction of ceria promoted by CNT. Ru is reduced at a low temperature of 160 °C in the Cs–Ru/CeO_2_ catalyst. When mechanically mixed with CNT (Cs–Ru/CeO_2_ + CNT_MM_), the Ru reduction temperature was further lowered to 140 °C. On the contrary, when Ru is supported on CNT or synthesized with Cs–Ru/CeO_2_ catalyst using coprecipitation (Cs–Ru/CeO_2_-CNT_Cp_) and hydrothermal (Cs–Ru/CeO_2_-CNT_Hy_) methods, Ru reduction temperature increased to the range of 200–250 °C, which is consistent with previous works on Ru/CNT catalyst.^33,34^ Furthermore, a second peak was observed at a temperature above 500 °C, which is attributed to the reduction of bulk lattice oxygen on the surface of cerium oxide, and the presence of CNT could further promote the reduction of ceria.^35^ The use of cerium oxide support plays a significant role in Ru reducibility due to its reversible oxidation state between Ce^3+^/Ce^4+^. The oxygen vacancies on the ceria surface strongly bind Ru species, forming a Ru–Ce–O bond, elucidating the low-temperature Ru reduction in comparison to CNT support.^10,36^ Based on the TPR data, mechanical mixing of CNT with the Cs–Ru/CeO_2_ catalyst further lowered the Ru reduction temperature associated with the unique characteristic of CNT. CNT improved the heating of the Cs–Ru/CeO_2_ catalyst, thus assisted in the reduction of Ru species from Ru^4+^ to Ru^0^ and improved the overall catalytic activity.

#### 3.2.4 TEM Measurement

**Figure 5:**
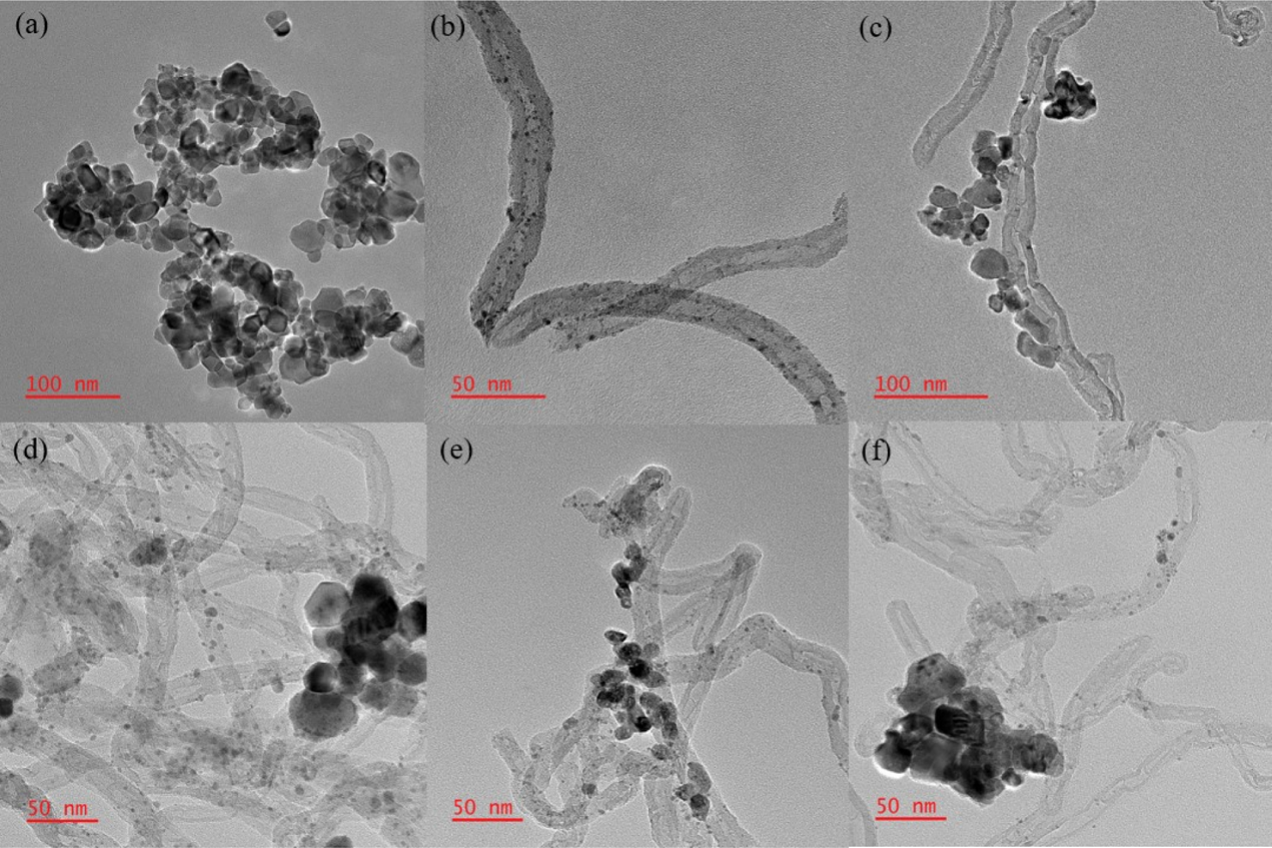
TEM images of (a). Cs–Ru/CeO_2_ (b). Cs–Ru/CNT (c). Cs–Ru/CeO_2_ + CNT_MM_ (d). Cs–Ru/CeO_2_-CNT_Cp_ (e). Cs–Ru/CeO_2_-CNT_Hy_ (f). Cs–Ru/CeO_2_ + Cs–Ru/CNT.

TEM imaging was utilized to reveal Ru particle size and dispersion in addition to the interaction between the Cs–Ru/CeO_2_ catalyst and CNT. As shown in Figure 5a, it is difficult to observe Ru species over the Cs–Ru/CeO_2_ catalyst, which is ascribed to the formation of the Ru–Ce–O phase, suggesting Ru species might be immersed in the ceria lattice, making it hard to be observed.^10^ Moreover, over CNT support, small and highly dispersed Ru species can be seen clearly, attributed to the high surface area of CNT.^32^ However, when CNT was mechanically mixed with Cs–Ru/CeO_2_ catalyst (Cs–Ru/CeO_2_ + CNT_MM_), Ru species was not spotted while CNT was dispersed close to the Cs–Ru/CeO_2_ catalyst, plausibly indicating a synergy among the constituents. The TEM image of the mechanically mixed catalyst (Cs–Ru/CeO_2_ + CNT_MM_) is consistent with the CO chemisorption results, signifying no considerable change in Ru particle size and dispersion as compared to the precursor Cs–Ru/CeO_2_ catalyst. Furthermore, when the Cs–Ru/CeO_2_-CNT catalyst was synthesized using hydrothermal (Cs–Ru/CeO_2_-CNT_Hy_) and coprecipitation (Cs–Ru/CeO_2_-CNT_Cp_) methods, Ru particles dispersed on the CNT was observed, whereas Ru species on cerium oxide support were very difficult to be seen. Based on the TEM images of Cs–Ru/CeO_2_-CNT_Cp_ and Cs–Ru/CeO_2_-CNT_Hy_ catalysts, the use of binary supports having different surface properties prompts severe non-uniform dispersion of Ru species on the surfaces of CNT and CeO_2_.^25,37^ Consequently, the non-uniform dispersion of Ru particles on the surface of CNT and CeO_2_ leads to a decrease in ammonia synthesis rate, while there is a great improvement of ammonia production rate on the combination of CeO_2_ and CNT supported Ru catalysts, as shown in Figure 5f and experimental data.

#### 3.2.5 Dielectric and Electrical Conductivity Measurements

**Figure 6:**
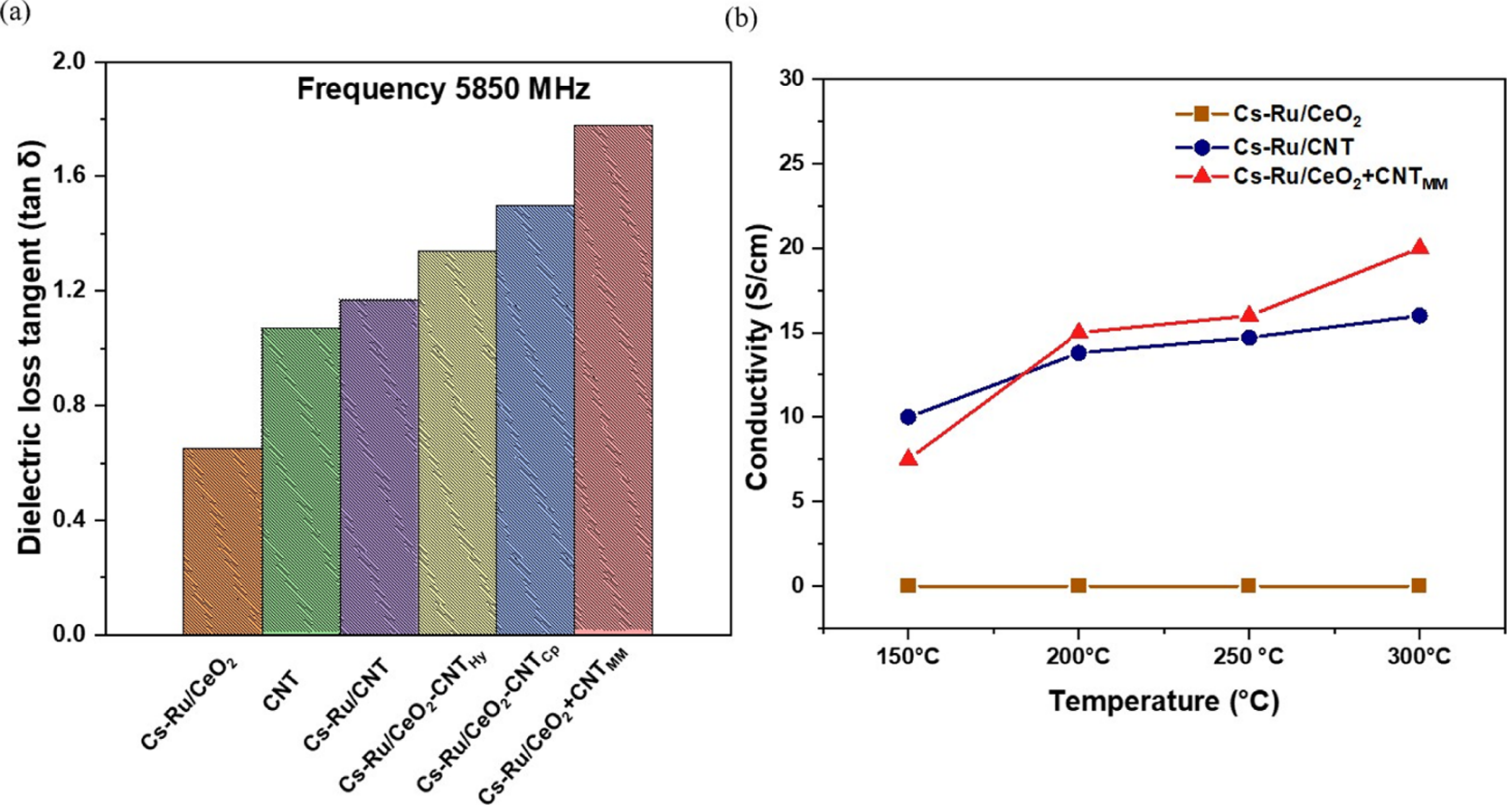
(a) Dielectric measurement for Cs–Ru/CeO_2_, Cs–Ru/CNT, Cs–Ru/CeO_2_ + CNT_MM_, Cs–Ru/CeO_2_-CNT_Cp_, and Cs–Ru/CeO_2_-CNT_Hy_ catalysts. (b). Electrical conductivity for Cs–Ru/CeO_2_, Cs–Ru/CNT, and Cs–Ru/CeO_2_ + CNT_MM_ catalysts.

Dielectric measurement was conducted to further study the effect of CNT on the Cs–Ru/CeO_2_ catalyst. All the tests were conducted at a reaction frequency of 5.850 GHz, and blank CNT was analyzed as well. Dielectric property is the characteristic of a material to convert the electromagnetic energy to heat and is commonly denoted as dielectric loss tangent. As shown in Figure 6a, blank CNT and CNT contained catalysts exhibited higher dielectric properties as compared to cerium oxide-supported catalyst. As reported in the literature and previous studies, carbon materials, including CNT, are very good microwave susceptors and can convert microwave irradiation to heat easily.^12,19,22^ The unique property of CNT relies on its excellent microwave absorbing capability and its ability to heat other materials indirectly or directly themselves as a catalyst.^24,27^ Furthermore, when CNT was mechanically mixed with the Cs–Ru/CeO_2_ catalyst (Cs–Ru/CeO_2_ + CNT_MM_), it further promoted the dielectric loss tangent of the Cs–Ru/CeO_2_ catalyst. This suggested the use of CNT contributed to improved microwave absorption and heating of the Cs–Ru/CeO_2_ catalyst, further enhancing the catalyst utilization and ammonia production. One of the main limitations regarding dielectric measurements is the magnetic effect. The magnetic component is not included in the measurement, a significant portion of the microwave irradiation. Magnetic and electrical components are the main constituents of microwave irradiation, and their exact contribution is not fully understood.^15^

Besides heating, CNT also has a unique advantage over metal oxide catalysts, including thermal and electrical conductivity.^31,38^ As shown in Figure 6b, the Cs–Ru/CeO_2_ catalyst exhibited inferior electrical conductivity as compared to the Cs–Ru/CNT catalyst at all temperatures studied. However, when Cs–Ru/CeO_2_ catalyst is mechanically mixed with CNT (Cs–Ru/CeO_2_ + CNT_MM_), it enhances the electrical conductivity of Cs–Ru/CeO_2_ catalyst, and as temperature increases, conductivity increases, facilitating electron transfer between support and promoter, expediting the catalytic activity toward ammonia production.^22,31,36,39^ We hypothesize that under microwave heating, electrical conductivity could further be enhanced.

#### 3.2.6 Thermal Imaging Measurement

**Figure 7:**
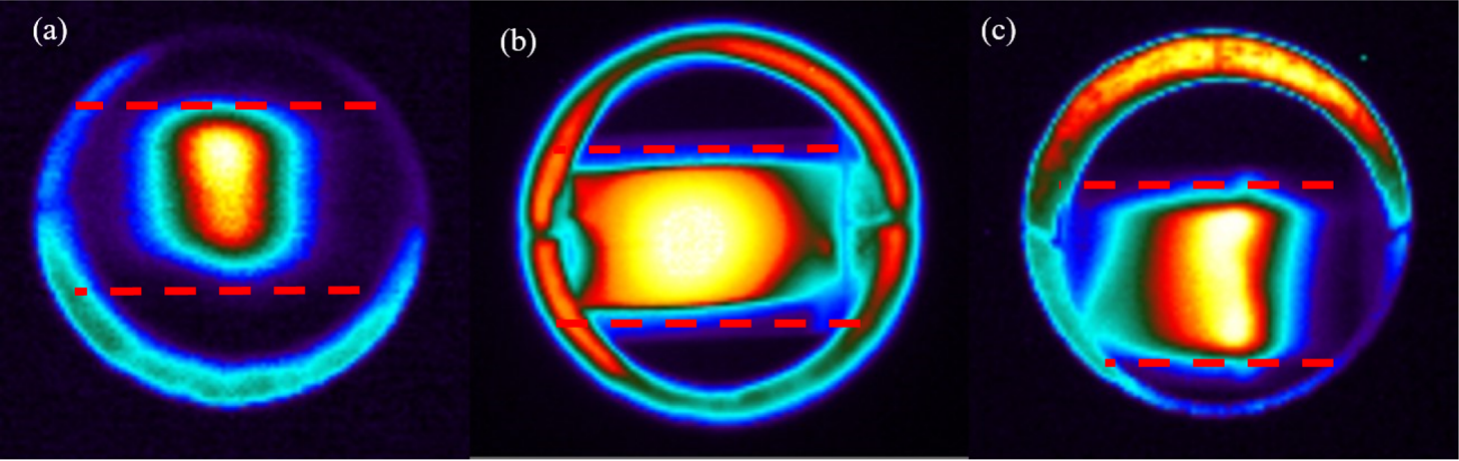
Thermal imaging for (a). Cs–Ru/CeO_2_ (b). Mechanical mixing (Cs–Ru/CeO_2_ + CNT_MM_) (c). Cs–Ru/CeO_2_ and CNT loaded adjacently (dashed lines indicate reactor direction).

To further understand the increase in catalytic activity and dielectric loss tangent for the mechanically mixed (Cs–Ru/CeO_2_ + CNT_MM_) catalyst, a thermal imaging camera was utilized to visualize microwave heating at a reaction temperature of 260 °C and a microwave frequency of 5850 MHz. The images shown in Figure 7b indicate that the use of CNT improved the heating of the Cs–Ru/CeO_2_ catalyst as compared to the precursor Cs–Ru/CeO_2_ catalyst presented in Figure 7a. Moreover, when Cs–Ru/CeO_2_ and CNT catalysts were loaded adjacently (i.e., the Cs–Ru/CeO_2_ layer and the CNT layer are contacted through the interfacial surface), CNT exhibited a higher heating rate as compared to its adjacent Cs–Ru/CeO_2_ catalyst and showed increased heating at the interface. This observation suggests that when Cs–Ru/CeO_2_ is mixed mechanically with CNT (Cs–Ru/CeO_2_ + CNT_MM_) at the particle level, there is an increased interfacial polarization between the many Cs–Ru/CeO_2_ and CNT particles that will lead to increased heating and ammonia production.^12,24,28^

## 4 Discussion

Microwave selective heating is fundamentally different from conventional heating, where incoming microwave irradiation interacts with the catalyst surface.^5,11,40^ Thus, electromagnetically susceptible catalyst design for microwave-initiated catalysis is crucial. In our previous publications, we have studied the Cs–Ru/CeO_2_ catalyst comprehensively and its interaction with microwave irradiation.^10,17^ In this study, the Cs–Ru/CeO_2_ catalyst mechanically mixed with CNT and the Cs–Ru/CeO_2_-CNT catalyst synthesized using coprecipitation and hydrothermal methods are investigated systematically. Moreover, a mixture of two Ru-based catalysts (Cs–Ru/CeO_2_ and Cs–Ru/CNT) with a 1:1 ratio is also studied. The mechanical mixture of Cs–Ru/CeO_2_ catalyst with CNT (Cs–Ru/CeO_2_ + CNT_MM_) exhibited superior activity as compared to the chemically synthesized, i.e., coprecipitation (Cs–Ru/CeO_2_-CNT_Cp_) and hydrothermal (Cs–Ru/CeO_2_-CNT_Hy_) catalysts. This superior catalytic performance is ascribed to CNT’s outstanding thermal and electrical properties and the plausible synergistic effect between CNT and the Cs–Ru/CeO_2_ catalyst.^22,38^ CNT are a very good microwave-susceptible material with wide applications in microwave-assisted heterogeneous catalysis.^12,14^ CNT can absorb microwaves efficiently and can convert electromagnetic energy to heat, thus compensating the heat limitation over the catalyst.^19,28^ In the mechanically mixed Cs–Ru/CeO_2_ + CNT_MM_ catalyst, heat generated from CNT can partially be supplied to Cs–Ru/CeO_2_, enabling it to reach the required temperature for catalysis to occur.^27^

As illustrated on the thermal imaging camera, when CNT was mechanically mixed with the Cs–Ru/CeO_2_ catalyst, improved heating was visualized on the catalyst bed as compared to the precursor catalyst, enhancing the catalyst utilization. During microwave heating, each consistent material reacts separately to the incoming microwave irradiation, and when mixed mechanically, interfacial polarization among the materials increases at the interface, plausibly resulting in a synergistic effect, subsequently improving the heating rate and catalytic activity.^12,22,24,28^ This improved interfacial polarization and synergistic effect further enhanced the reduction of ceria and Ru, boosting ammonia production. Jie et al.^24^ investigated the physical mixing of Fe and activated carbon (AC) under microwave irradiation for hexadecane dehydrogenation. According to their findings, the physical mixing of Fe with AC improved the heating effect under microwave and ascribed to the synergistic effect resulting from increased interfacial polarization between Fe and AC species.^24^

Besides heating, the high electrical conductivity of CNT also enhanced electron transfer from the promoter and support to the Ru surface, facilitating N_2_ dissociation and ammonia production.^10,36^ The rate-determining step in ammonia synthesis is N_2_ dissociation, and increasing electron density on the surface of Ru expedites N_2_ bond cleavage, resulting in enhanced ammonia production.^41^ Cs–Ru/CeO_2_ catalyst forms a Ru–Ce–O phase, which facilitates electron transfer from CeO_2_ support and Cs promoter to the Ru surface, and when CNT was mixed with the Cs–Ru/CeO_2_ catalyst, it further eased electron transfer between support and promoter, attributed to its high electrical conductivity.^10,29,31^ However, lower activity is observed in the chemically synthesized catalyst even though the same constituent was used. The low catalytic activity is associated to a change in the electronic and geometric properties of the Cs–Ru/CeO_2_ catalyst, modifying Ru particle size and dispersion. Even though high dispersion and small Ru particle is analyzed on the chemically synthesized catalysts, i.e., Cs–Ru/CeO_2_-CNT_Cp_ and Cs–Ru/CeO_2_-CNT_Hy_, they exhibited inferior catalytic performance. The low activity on the binary support is designated to surface property difference, resulting in non-uniform dispersion of Ru species over the CeO_2_ and CNT, reducing the number of active sites for N_2_ and H_2_ dissociation, thus lowering the overall catalytic activity toward ammonia production.^37^ A similar discovery is reported by Xu et al.^25^ on the combination of Ru-based catalysts (K–Ru/MgO and K–Ru/CNT) and K–Ru/MgO-CNT catalysts for ammonia synthesis. Based on their investigation, the combination of K–Ru/MgO and K–Ru/CNT catalysts exhibited higher catalytic activity toward ammonia synthesis as compared to K–Ru/MgO-CNT. The high catalytic performance on the combination of the Ru-based catalyst is related to complementary interaction, while the low catalytic activity for K–Ru/MgO-CNT is ascribed to the non-uniform dispersion of Ru species on CNT and MgO, related to surface property differences.^25^

The ammonia synthesis for the combined Ru-based catalyst is much higher than that of the individual Cs–Ru/CNT and Cs–Ru/CeO_2_ catalysts. The high ammonia production rate for the combined Ru-based catalyst could be related to the combination of the high graphitization of CNT and the basicity of CeO_2_.^42^ In the mixture catalysts (i.e., Cs–Ru/CeO_2_ and Cs–Ru/CNT), CeO_2_ can enhance the basicity of the Cs–Ru/CNT catalyst and resolve the electron-withdrawing nature of CNT, thus elevating the catalytic activity for ammonia synthesis.^25,42^ At the same time, Cs–Ru/CNT can facilitate the transfer of electrons from alkali (CeO_2_) to the Ru surface effortlessly for the Cs–Ru/CeO_2_ catalyst.^25^ Besides that, both mechanically combined Cs–Ru/CeO_2_ and Cs–Ru/CNT catalysts kept their intuitive catalytic activity toward ammonia synthesis. However, when combined due to their complementary interactions, the limitations of Cs–Ru/CeO_2_ and Cs–Ru/CNT catalysts can be reduced to some level when both catalysts are combined.^25^

In addition, we found that when Cs–Ru/CeO_2_ and CNT nor Cs–Ru/CeO_2_ + Cs–Ru/CNT are present as a mechanical mixture and exposed to microwave irradiation simultaneously, each catalyst material will respond to the incoming microwave irradiation independently, depending on their dielectric properties. This interaction between the microwave and the catalysts raises possible synergistic effects of heating as the mechanically mixed catalysts are exposed to the microwave independently.^12,24^ This phenomenon of microwave heating, when mixed mechanically, initiated interfacial polarization at the surface, causing an increase in local electric field strength, creating heating differences among the constituents, and improving the overall microwave heating of the catalyst system.^22,24,28^ The images obtained from the thermal imaging camera elucidated this phenomenon. In the mechanically mixed CNT with Cs–Ru/CeO_2_ catalyst, the synergistic effect due to improved heating and electrical conductivity also facilitated the transfer of electrons to the Ru surface, assisting N_2_ bond cleavage as N_2_ dissociation is the rate-determining step in ammonia synthesis and enhanced the overall catalytic activity for ammonia synthesis.^10,24,27,43^

In our comprehensive study on the effect of CNT over Cs–Ru/CeO_2_ catalyst based on experimental data coupled with various characterization techniques including thermal imaging camera, electrical conductivity, dielectric measurement, and H_2_-TPR, the heat limitation of Cs–Ru/CeO_2_ catalyst is optimized when mechanically mixed with CNT. Besides improved microwave heating, the high electrical conductivity of CNT also enhanced electron transfer from the CeO_2_ support and Cs promoter to the Ru surface, expediting N_2_ dissociation over the Cs–Ru/CeO_2_ catalyst and increased the overall catalytic activity toward ammonia synthesis. We hypothesize that under microwave irradiation, all these conditions could further be enhanced.

## 5 Conclusions

Ammonia is a viable H_2_ energy carrier for future clean energy when produced from renewable energy sources to transition away from regular fossil fuel energy. The development of microwave technology together with microwave susceptible catalyst design can produce the desired ammonia product under mild conditions and can operate with the supply of intermittent renewable energy sources. In this study, we have demonstrated that mechanical mixing of the Cs–Ru/CeO_2_ catalyst with CNT increased the ammonia production from 535 to 1822 μmol NH_3_/g_cat_ h. This increase in catalytic performance is ascribed to enhanced microwave heating coupled with improved electrical conductivity and dielectric property, facilitating N_2_ dissociation and subsequently in higher ammonia production. Besides mechanical mixing of CNT, the Cs–Ru/CeO_2_-CNT catalyst was chemically synthesized using hydrothermal and coprecipitation methods. The chemically synthesized catalysts showed inferior activity toward ammonia synthesis. The low catalytic activity was ascribed to the non-uniform dispersion of Ru particles on the binary CeO_2_ and CNT support, reducing electron transfer from the CeO_2_ support and Cs promoter to the Ru surface, resulting in lower N_2_ dissociation and ammonia production, while in the mechanically mixed catalyst, mixing of CNT further eased electron transfer between support and promoter, enhancing the ammonia production. Not only mechanical mixing of Cs–Ru/CeO_2_ with CNT enhanced the catalytic activity, but also the combination of two Ru-based catalysts, i.e., Cs–Ru/CeO_2_ and Cs–Ru/CNT mixed in a 1:1 weight ratio, also displayed higher catalytic activity as compared to their individual activity. A thermal imaging camera was utilized to visualize the heating rate under microwaves and elucidate the improved catalytic activity. Images from the camera indicated an increase in microwave heating rate when CNT was mixed mechanically with the Cs–Ru/CeO_2_ catalyst. We believe this study may open new avenues in developing advanced microwave-sensitive catalysts for ammonia synthesis.

## Supporting Information Available

Additional experimental details and characterization on material ratio, Ru as an active site, and SEM images (PDF)
