# Supplementary material for: Insight into Enhanced Microwave Heating for Ammonia Synthesis: Effects of CNT on the Cs–Ru/CeO2 Catalyst
Source: ACS Appl Mater Interfaces. 2023 May 11;15(20):24296–305. doi: 10.1021/acsami.3c00132 (PMC10214378; doi:10.1021/acsami.3c00132)
Supplement: Supplementary file 1 — am3c00132_si_001.pdf [file am3c00132_si_001.pdf]

Supporting Information

# Insight into Enhanced Microwave Heating for Ammonia Synthesis: Effects of CNT on Cs-Ru/$CeO_2$ Catalyst

Alazar Araia, Yuxin Wang, Changle Jiang, Sean Brown, Ashley Caiola, Brandon Robinson, Wenyuan Li, Jianli Hu *

Department of Chemical and Biomedical Engineering, West Virginia University, Morgantown, USA
*Corresponding Author: john.hu@mail.wvu.edu

## SEM Images

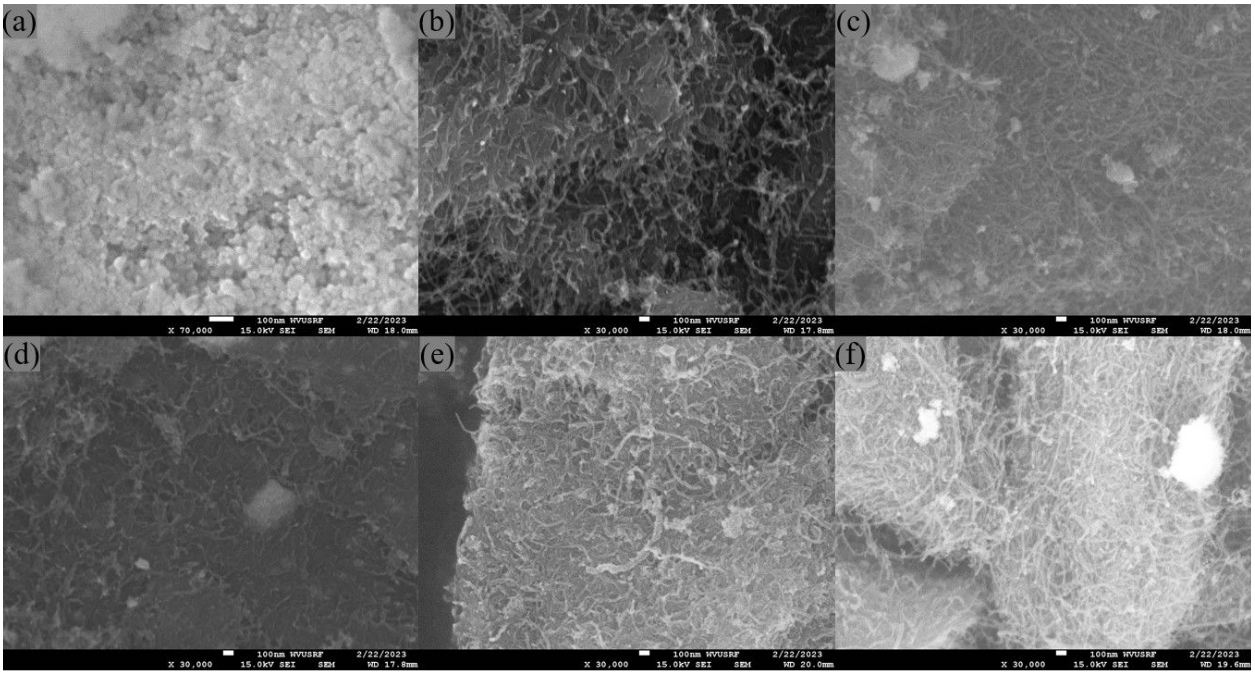


**Figure S1.** SEM images of (a). Cs-Ru/$CeO_2$ (b). Cs-Ru/CNT (c). Cs-Ru/$CeO_2$+$CNT_{MM}$ (d). Cs-Ru/$CeO_2$-$CNT_{Cp}$ (e). Cs-Ru/$CeO_2$-$CNT_{Hy}$ (f). Cs-Ru/$CeO_2$+Cs-Ru/CNT

SEM images was used to study the effect of CNT on Cs-Ru/$CeO_2$ and shown in Figure S1. Based on the SEM images it is very difficult to detect Ru or Cs species in all the catalysts. However, we can clearly see $CeO_2$ and CNT support in Figure S1 a and S1 b. Moreover, in the mechanically mixed catalyst shown in Figure S1 c, we can see CNT dispersed close to Cs-Ru/$CeO_2$ catalyst. In the chemically synthesized catalysts, shown in Figure S1 d and S1 e, we can distinctly see CNT supports while $CeO_2$ is highly dispersed on CNT making it hard to visualize. Cs-Ru/$CeO_2$+Cs-Ru/CNT catalysts are spread close to each other as shown in Figure 1S f.

**Material ratio of Cs-Ru/$CeO_2$ catalyst to CNT**

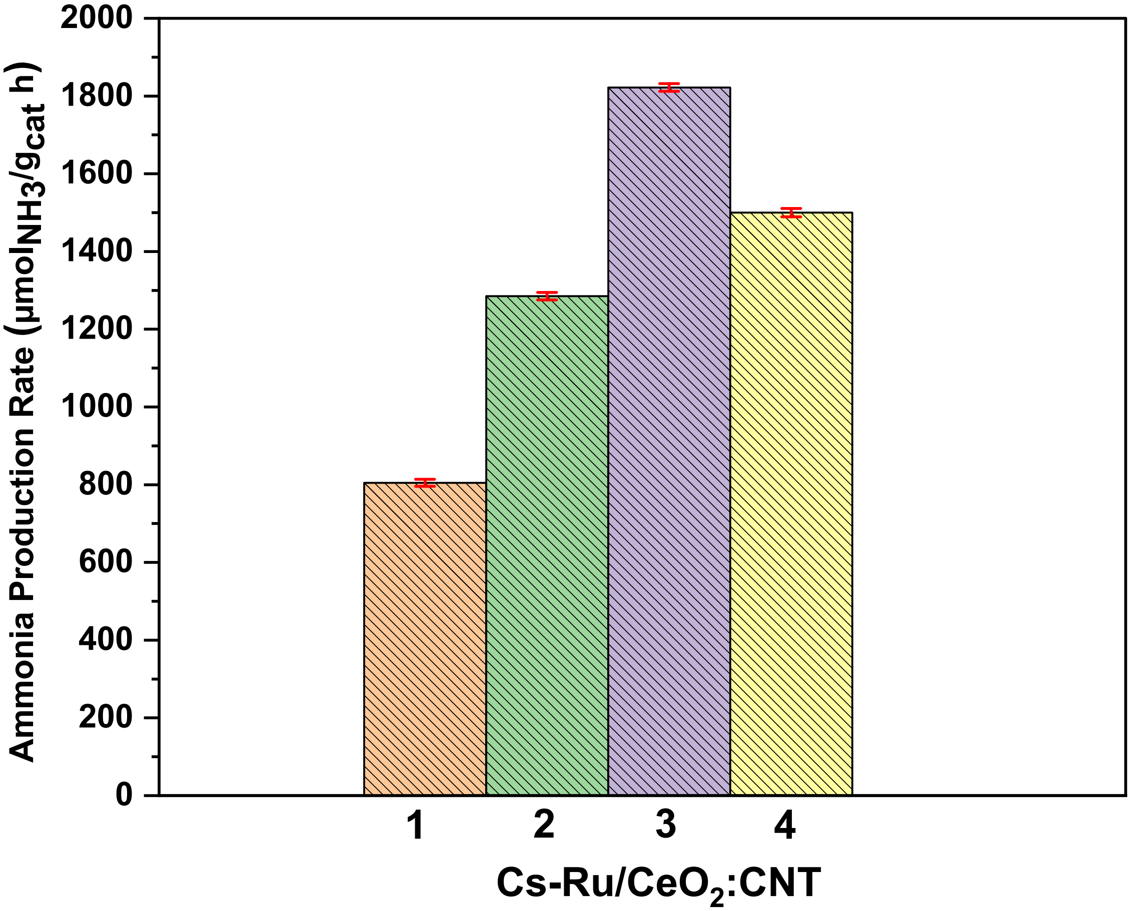


**Figure S2.** Material ratio of Cs-Ru/$CeO_2$ catalyst to CNT

Mechanically mixed Cs-Ru/$CeO_2$+CNT catalyst with varying Cs-Ru/$CeO_2$ catalyst to CNT material ratio is studied at 260 °C under microwave reactor and shown in Figure S2. Based on our study, 3:1 material ratio of Cs-Ru/$CeO_2$ to CNT exhibited the highest ammonia production rate of 1822 µmol $NH_3/g_{cat}$ h. A material ratio of 1:1 showed the lowest ammonia production rate of 805 µmol $NH_3/g_{cat}$ h while a ratio of 2:1 Cs-Ru/$CeO_2$ to CNT exhibited 1285 µmol $NH_3/g_{cat}$ h ammonia production rate. Moreover, the highest material ratio of 4:1 Cs-Ru/$CeO_2$ to CNT showed ammonia production rate of 1500 µmol $NH_3/g_{cat}$ h. Based on the experimental data, we concluded the optimum ratio of Cs-Ru/$CeO_2$ to CNT is 3:1 with superior ammonia production rate.

**Active site over $Cs\text{-}Ru/CeO_2$ catalyst**

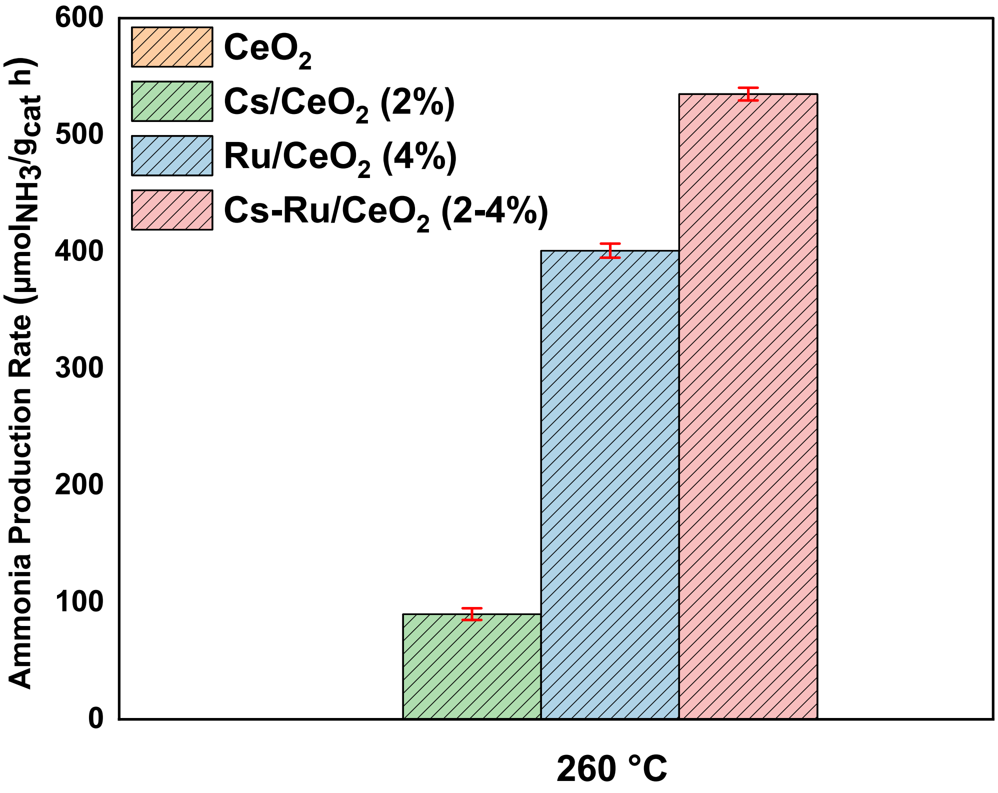


**Figure S3**. $CeO_2$, $Cs/CeO_2$ (2 wt.%), $Ru/CeO_2$ (wt.4%) and $Cs\text{-}Ru/CeO_2$ (2-4 wt.%) for microwave-assisted ammonia synthesis at 260 °C

All the constituents of $Cs\text{-}Ru/CeO_2$ catalyst loaded with 2 wt.% Cs and 4 wt.% Ru are studied to determine Ru is the active site. Based on our findings and shown in Figure S3, $CeO_2$ support exhibited no catalytic activity towards ammonia synthesis and no peak was observed in the bar graph. $Cs/CeO_2$ (2 wt.%) catalyst showed 90 μmol $NH_3/g_{cat}$ h ammonia production rate and $Ru/CeO_2$ (4 wt.%) exhibited 401 μmol $NH_3/g_{cat}$ h ammonia production rate suggesting Ru is the active sites for ammonia synthesis. Moreover, when Cs was used as promoted and $CeO_2$ as support for Ru catalyst the activity increased to 535 μmol $NH_3/g_{cat}$ h.
